# Progress in Renal Medicine

**Published:** 1901-01-19

**Authors:** 


### PROGRESS IN RENAL MEDICINE.

(*Continued from page* 249.)

**Cyclic Albuminuria.**—G. A. Sutherland[19] combats the view that this is a result of previous nephritis, and points out that other evidences of nephritis, such as casts, are absent, and that the albumen itself vanishes from time to time. It has been ascribed to the effects of oxaluria, masturbation, altered blood states, circulatory and vaso-motor changes, and the uric acid diathesis. The writer thinks that there is a toxæmic state similar to or identical with the last-named one, and compares the condition to the intermittent albuminuria seen in Grave's disease, where, too, a poison probably circulates in the blood. The patients are generally nervous and listless, and complain of pain in the head, limbs, and back. The pulse is rapid and of low tension, there is occasional slight œdema, and the skin is apt to flush. The urine is free from albumen so long as the patient remains in bed, but reappears when he gets up, though in other respects it remains quite normal. We must distinguish from the cyclic form (*a*) albuminuria after a meal or certain kinds of food irrespective of position, and (*b*) that following severe exertion, such as racing. A duration for several years has often been noted, and in fifteen cases of his own the latest examinations showed no symptoms of the development of true nephritis. On the whole, he finds no evidence that the disease is an early stage of, or tends to nephritis, but there are few or no facts known as to the final history of these patients. Dyce Duckworth's rule as to passing them for public services is that the albumen must disappear altogether, that it must not be due to scarlatina, and that there must be no evidence of renal or cardiac change. Until our knowledge is more complete, these and similar rules for insurance must probably be carried out. Sutherland regards these patients as neurotic, and advises for them a quiet healthy life, such as would be best for the subjects of chorea, free from anxiety and strain.

**Uræmia.**—H. P. Coile[20] advances a singular view as to this condition which perhaps is not without some foundation. He thinks that it occasionally appears without structural disease of the kidney, or suppression or obstruction of the urinary flow. Indeed it may be found when the urinary excretion of urea is above the normal, if from any cause the disintegration of the tissues is excessive so that the blood is overloaded. If much nitrogenous food is then taken and cannot be assimilated the products of its destruction accumulate. Active diaphoresis in such cases is of little value as it removes much water and little urea from the blood. Thus sweat only contains one five hundred and thirty-fifth part of the urea contained in an equal quantity of urine. Stimulation of the kidneys and active catharsis will therefore be our best remedies together with abstinence from much food, especially from nitrogenous articles of diet. He gives an interesting case where uræmia set in when the kidneys and other organs were normal and neither albumen or sugar were present, though the specific gravity was as much as 1035. F. Evill and Samuel West[21] report cases of acute albuminuria where severe uræmia appeared, two of which recovered entirely without a trace of albumen and a third died. In all of them the albuminuria came on very suddenly with blood casts and great diminution of urine, and the patients quickly passed into a renal toxæmic state. West remarks that such instances are very rare, except in the course of fevers, such as scarlatina, which are usually fatal. There was no such cause in these patients except perhaps some recent influenza.

**Urinary Pigments.**—Some of these are true pigments, others are bodies, chromogens, which become coloured under the influence of air and light. Some are taken in with food or medicine, some are normal constituents of the urine, and others appear in it only under morbid conditions. A. E. Garrod[22] discusses another class which are found in traces during health, while some of them appear in excess in disease. These include urobilin, urochrome, hæmatoporphyrin, and uroerythrin. Urobilin is formed chiefly in the large intestine, and even that which is present in bile is part of that which has been absorbed from the intestine and is not due to formation in the liver. The latter organ has, of course, considerable influence on its formation, whether by influencing its production in the intestine, or by destroying some portion of it after it has been absorbed. It has also been asserted that urobilin may be formed by the destruction of blood cells, but this point has not yet been decided. Excess of urobilin in the urine is common in fevers and hepatic diseases, and where hæmolysis is present, such as pernicious anæmia, where, indeed, it forms a useful sign of the progress of the case. Hæmatoporphyrin is either an intermediate or a by-product of metabolism, and probably disorders of the liver are the chief causes which bring about its appearance in excess. Such are the fatty degenerations caused by heart disease, phthisis, lead poisoning, and the effects of gout and fevers. The port-wine colour of some of these urines, and especially that in sulphonal poisoning are not wholly due to the excessive hæmatoporphyrin they contain, but to another unknown pigment which accompanies it. Intestinal hæmorrhages have been thought to be a cause especially in sulphonal poisoning, but frequently they cannot be demonstrated.

Urochrome and uroerythrin have been but little investigated either as to their origin or importance. It is to the latter that the pink colour of "brickdust" uratic deposits is due. It is of course seen in health, but is even more common in febrile and hepatic conditions. The presence of indican in the urine has been thought to have some relation to the amount of HCl. produced in the stomach, but A. A. Jones has investigated this in 162 cases, and reports that no constant relation exists between them.[23] It is clear that indican is derived from the decomposition of albuminous substances in the small intestines. This putrefaction cannot, it seems, be prevented by the HCl of the stomach. Indeed, indican may be excessive in gastric ulcer where a large amount of free acid is often present. In carcinoma the acid may be present or not, but usually indican is excessive. On the whole, the writer thinks that if albuminous digestion does go on in the intestine indican will be absent, whether or no free acid is found in the stomach. Putrefaction may thus be hindered by the combined chlorides, by the bile, by the succus entericus, and other means. On the other hand, where the intestine is paralysed by ileus indican is present in large quantities. *Urotropin* is becoming more and more used both for cystitis and bacterial affections of the urinary tract, and on account of its power of dissolving uric acid at the temperature of the body. Nicolaier,[24] who first introduced it into practice, points out that its presence in the urine is shown on the addition of bromine water, by an orange precipitate, which dissolves on heating. A few drops of a saturated solution of bromine give this test in urine containing urotropin. He considers that the drug has a solvent action on uric acid calculi, but holds that not more than 20 or 25 grains a day should be administered. This may be continued for an indefinite period.

**The Smegma Bacillus.**—O. A. Dahms[25] thinks that this organism should be distinguished from the bacillus of syphilis, which latter is rather larger, more slender, less readily decolourised by alcohol, but loses colour in acids in thirty to forty seconds. Moreover it stains well by Doutrelepont's method, while the smegma bacillus does not. The tubercle bacillus, besides retaining its stain in strong alcohol for five minutes, takes a bright red with the stain called Sudan III., which has no effect on the smegma variety. T. H. Bryant[26] discusses the nature of essential hæmaturia, and shows the large number of affections which must be excluded to arrive at a diagnosis. Still it is quite clear that many such cases do occur in persons otherwise in good health. He would regard them as caused by an abnormal or varicose renal papilla, such as has been actually found in two cases by Fenwick. It is possible that some instances are due to an angio-neurosis following a severe shock. However, it is remarkable that some of these patients have recovered completely after nephrotomy though nothing whatever abnormal was found in the kidney. The condition has been well compared to epistaxis in a healthy individual.

[19] Clin. Jour., Sept. 7. [20] Merck's Archives, May. [21] Lancet, Oct. 6. [22] Lancet, Nov. 10. [23] New York Med. Jour., April 28. [24] Med. Chron., May. [25] Med. Rec., May 5. [26] Clin. Jour., July 25.

